# Diabetes and postoperative cognitive dysfunction and delirium in adults: mechanisms, biomarkers, and clinical management

**DOI:** 10.3389/fendo.2026.1745202

**Published:** 2026-03-16

**Authors:** Abdulrahman Khaled Alwesabi, Yuhu Ma, Boxiong Gao, Jinxiang Xie, Ji chengying, Su Xiaodong, Qian Fu, Ying Liu, Qijing Liu, Jiayi Xie, Bokang Yang, Chaohui Gao, Yatao Liu

**Affiliations:** 1 The First School of Clinical Medicine, Lanzhou University, Lanzhou, Gansu, China; 2 Department of Anesthesiology and Surgery, First Hospital of Lanzhou University, Lanzhou, Gansu, China

**Keywords:** biomarkers, diabetes mellitus, neuroinflammation, postoperative cognitive dysfunction, postoperative delirium

## Abstract

Postoperative cognitive dysfunction (POCD) and postoperative delirium (POD) are common perioperative neurocognitive disorders, particularly affecting individuals with diabetes, who show a disproportionately higher susceptibility. Diabetic patients are at higher risk due to blood sugar fluctuations, vascular changes, and inflammation that can affect brain function. This review explores how diabetes contributes to POCD and POD, the role of biomarkers in identifying those at risk, and strategies to prevent and manage these complications. A thorough analysis of current studies highlights that factors such as hyperglycemia, glycemic variability, and diabetes-related complications significantly increase the likelihood of cognitive problems after surgery. While several tools exist to assess cognition and delirium, none reliably detect early changes on their own, underscoring the need for integrated approaches that combine biomarkers and clinical assessment. Interventions like tight blood sugar control, careful perioperative monitoring, and cognitive rehabilitation may help reduce these risks. Overall, understanding the link between diabetes and postoperative cognitive complications and implementing personalized care plans are key to improving recovery and quality of life for diabetic patients. Future research should prioritize the standardization of diagnostic criteria, the clinical validation of perioperative biomarkers, and the development of targeted preventive and therapeutic strategies for patients at increased perioperative neurocognitive risk.

## 1 Introduction

Following surgery, older adults and individuals with diabetes are at increased risk of perioperative neurocognitive disorders, particularly postoperative delirium (POD), characterized by acute and fluctuating disturbances in attention and consciousness, and postoperative cognitive dysfunction (POCD), which involves more prolonged impairments in memory and executive function (1, 2). These complications can prolong hospitalizations and worsen long-term outcomes. Diabetes may heighten susceptibility to POD and POCD through chronic hyperglycemia, vascular injury, autonomic dysfunction, and impaired cerebral metabolic resilience, which reduce the brain’s ability to tolerate perioperative stress (3). Recognizing these risks is essential for early identification and management, which can improve recovery and prevent long-term cognitive decline (4). POD generally arises between hours to days following surgery, presenting as an abrupt and variable impairment in attention and consciousness, frequently resolving within days to weeks. Conversely, POCD entails a more nuanced and enduring deterioration in memory, attention, and executive function, potentially lasting from weeks to months. Major operations, including gastrointestinal, orthopedic, or cardiac procedures, can precipitate these complications, particularly in patients with diabetes due to their pre-existing metabolic and vascular vulnerabilities (5). Large observational and cohort studies have quantified this increased risk, demonstrating that diabetes is associated with approximately a 1.3–2.0-fold higher incidence of postoperative delirium and postoperative cognitive dysfunction compared with non-diabetic patients, with risk magnitude influenced by age, surgical type, and perioperative glycemic control (1, 6). Poor long-term glycemic control, reflected by elevated HbA1c, and perioperative hyperglycemia have consistently been identified as independent predictors of postoperative neurocognitive complications. Nevertheless, the majority of existing evidence is observational, and residual confounding from comorbidities, baseline cognitive status, and perioperative variables constrains clear causal inference.

Prospective studies are still needed to provide standard risk levels and routes.

Complicating diagnosis and treatment are comorbidities like pre-existing cognitive impairment, microvascular disease, neuropathy, and hypoglycemia, which may mask or mimic POCD and POD symptoms. Early detection of these abnormalities is essential to enhance surgical care, alleviate cognitive decline, and facilitate long-term recovery (7). Validated diagnostic tools differentiate these entities: CAM and DRS are commonly used for the assessment of POD, exhibiting excellent sensitivity and specificity, whereas MMSE and MoCA are used for screening for POCD, despite the possibility of overlooking modest abnormalities (8). Emerging techniques, including EEG, ERP, PET, and fMRI, along with biomarkers of inflammation and oxidative stress, provide mechanistic insights and may enhance early detection (9). Although these advanced approaches have great potential, their complexity, cost, and variability of postoperative care make them not generally used in clinical practice. Recent advances in AI and machine learning offer non-invasive, cost-effective approaches for early detection and risk stratification of POD and POCD in diabetic patients. AI-driven cognitive evaluations, predictive analytics, and automated screenings could enhance individualized perioperative management by incorporating patient-specific metabolic, vascular, and cognitive risk factors. (4). This review synthesizes current evidence on the mechanisms, biomarkers, and management strategies for POD and POCD in adults with diabetes, highlighting validated diagnostic instruments, emerging biomarker-driven approaches, and innovative AI-supported risk-prediction tools. By integrating mechanistic understanding with clinical frameworks, it aims to identify research gaps and support evidence-based strategies to optimize perioperative neurocognitive outcomes in this high-risk population (10).

This article is presented as a narrative review synthesizing current evidence on the mechanisms, biomarkers, and perioperative management of postoperative cognitive dysfunction (POCD) and delirium (POD) in adults with diabetes. Relevant literature was identified through searches of major scientific databases, including PubMed and Google Scholar, using combinations of keywords such as diabetes, postoperative delirium, postoperative cognitive dysfunction, neuroinflammation, and biomarkers. Priority was given to recent clinical studies, systematic reviews, and key experimental research addressing mechanistic pathways and clinical implications. As a narrative synthesis, this review does not follow a formal systematic review protocol but aims to provide an integrative overview of this evolving field.

## 2 The pathophysiology of postoperative cognitive dysfunction and delirium

### 2.1 Mechanisms of postoperative cognitive dysfunction

Particularly in diabetic patients, postoperative cognitive dysfunction (POCD) is mostly associated with neuroinflammation. One aspect of diabetes is chronic inflammation, which increases the brain’s sensitivity in patients with diabetes strong inflammatory reaction following surgery. Pro-inflammatory cytokines, including IL-6, TNF-α, and IL-1β, are produced in response to surgical damage during the perioperative period (11). By passing the blood-brain barrier (BBB), these cytokines can activate microglia, the brain’s innate immune cells. This stimulation triggers an inflammatory response that disrupts normal brain function and causes cognitive problems. For patients with diabetes, raised baseline inflammation brought on by underlying metabolic dysregulation aggravates their illness and increases their vulnerability to additional brain injury. Important processes for learning and memory, neuroinflammation disturbs synaptic plasticity and neurogenesis. Therefore, patients with diabetes are more prone to get POCD following surgery (12). Moreover, inflammatory mediators disrupt neurotransmitter systems, including dopamine, serotonin, and glutamate, all of which are vital for cognitive function (13). Hyperglycemic states, frequent in patients with diabetes, aggravate neuroinflammation in POCD by inducing oxidative stress and advanced glycation end-products (AGEs), thereby triggering immunological responses and activating immune receptors. These inflammatory mediators greatly influence microglia in the brain, which are vital for immune monitoring in the central nervous system (CNS) (14). Chronic low-grade neuroinflammation reduces the capacity of neurons to adjust to cognitive tasks, therefore affecting executive function and memory consolidation. Extensive studies on the molecular etiology of POCD and potential preventive strategies have been conducted. The molecular mechanisms by which sevoflurane induces POCD may provide insight into treatment and prevention strategies (15). Animal models used in clinical research help clarify POCD diagnosis criteria and the processes involved in sevoflurane-induced POCD, potentially improving preventive and treatment approaches for the disorder (16).

**Figure 1:**
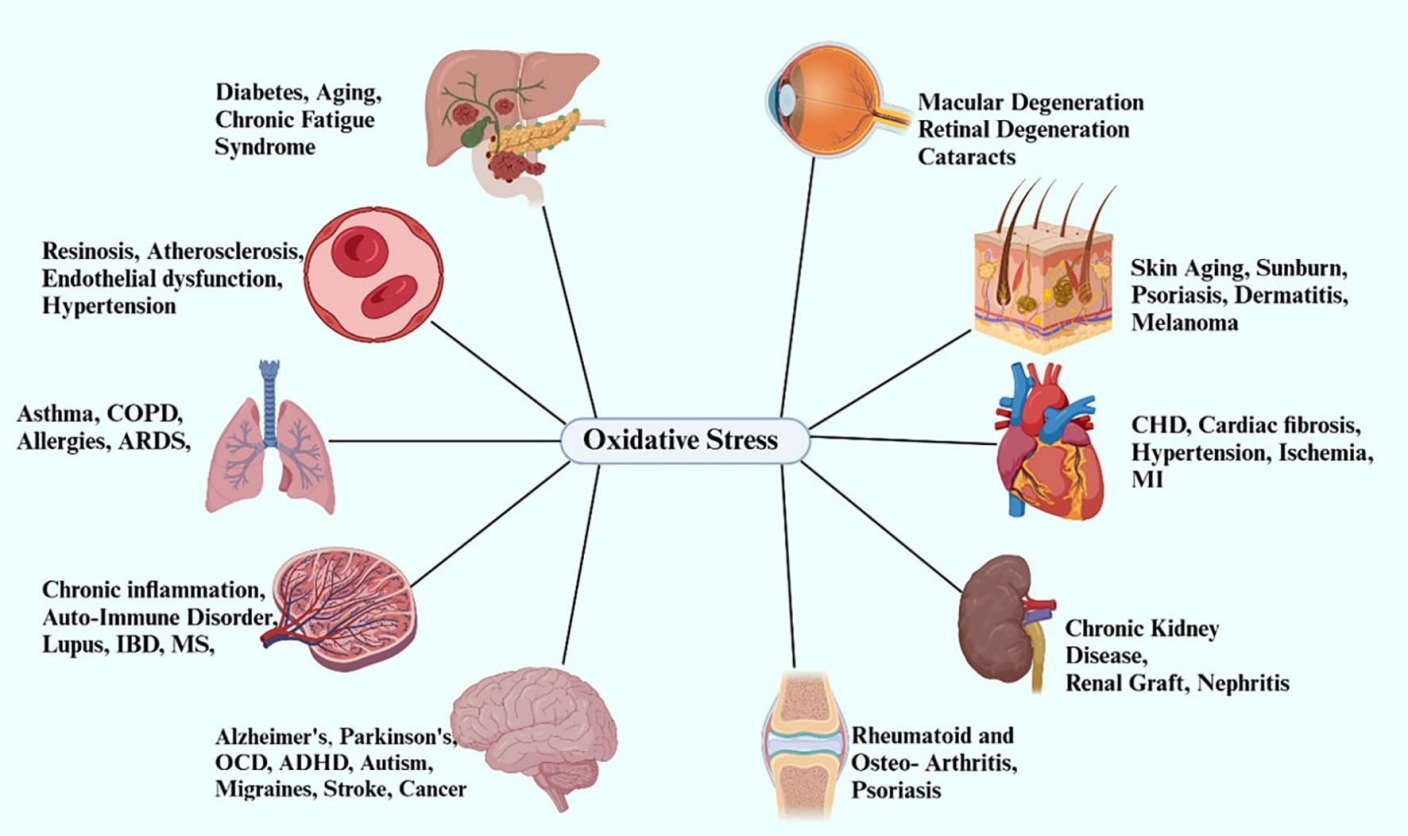
The influence of oxidative stress on multiple organs and systems, particularly in individuals with diabetes. It underscores the role of oxidative stress in the development of complications such as diabetic retinopathy, nephropathy, and cardiovascular diseases, positioning it as a critical contributor to the progression of diabetes-related pathological conditions.

Additionally, another study explored the inflammatory processes of the CNS, highlighting how changes in peripheral circulation and pathogenic interactions between peripheral circulation and the CNS could exacerbate POCD. These efforts aim to enhance the understanding of POCD’s onset, development, and effective preventive measures. For those with diabetes, POCD is largely driven by neurodegeneration. Persistent hyperglycemia and insulin resistance increase neurodegenerative processes in the brain, making individuals with diabetes more likely to experience cognitive impairment after surgery (4). Elevated oxidative stress and inflammation in patients with diabetes promote the formation of amyloid plaques and tau protein phosphorylation, characteristics of neurodegenerative diseases like Alzheimer’s. These processes lead to synaptic malfunction and cell death in regions of the brain crucial for cognitive functions, such as the hippocampus and prefrontal cortex (17), **Figure 1**.

Evidence supporting the role of neuroinflammation and oxidative stress in POCD is derived primarily from well-established animal models and observational human studies, whereas direct causal links between diabetes-related neurodegeneration (e.g., amyloid-β accumulation and tau hyperphosphorylation) and POCD in surgical patients remain emergent and are supported mainly by experimental and translational data (18). Surgery and anesthesia accelerate neurodegenerative changes by raising oxidative stress, causing mitochondrial dysfunction, and disrupting pro-survival and pro-apoptotic signaling pathways inside neurons. These factors contribute to cognitive decline typical of POCD by promoting cellular death and weakening synaptic connections (19). Moreover, oxidative stress exacerbates diabetes-related neurotoxicity, further emphasizing the need for early identification and intervention in this at-risk population. In patients with diabetes, poor glucose metabolism causes excess glucose to combine with oxygen, generating reactive oxygen species (ROS), which damage proteins, lipids, and DNA, impairing neuronal integrity. This oxidative damage accelerates neurodegenerative processes, especially in sensitive brain regions such as the prefrontal cortex (important for executive functioning) and the hippocampus (vital for memory storage) (20).

Neurodegeneration in POCD is also linked to the accumulation of improperly folded proteins like tau and amyloid β. This results in defective neural connections, leading to executive cognition and memory deficits. A study evaluating the correlation between postoperative delirium (POD) and POCD found that neurovascular changes are often associated with the development of both conditions (21). High HbA1c readings, which indicate poor glycemic control, may increase the risk of POCD in patients with diabetes, highlighting the need for further research on how glycemic management impacts POCD risk in both diabetic and non-diabetic patients. Diabetes complicates POCD due to its co-occurrence with vascular abnormalities, chronic inflammation, and neurodegenerative alterations. These factors increase the brain’s vulnerability to the physiological and metabolic demands of anesthesia and surgery (1).

While these hormones are part of the body’s adaptive stress response, excessive levels can negatively affect cognitive function. In patients with diabetes, insulin resistance and hyperglycemia elevate pro-inflammatory cytokines such as TNF-α and IL-6, further promoting neuroinflammation and cognitive decline (22). Additionally, hypotension and hypoxia during anesthesia can compromise cerebral perfusion, increasing the risk of ischemic injury to sensitive brain regions, such as the hippocampus, which is crucial for memory and learning. Anesthetic drugs and perioperative stress create a vicious cycle that exacerbates cognitive loss in patients with diabetes, as their pre-existing vascular dysfunction, inflammation, and metabolic abnormalities make the brain more sensitive to anesthesia-induced neurotoxicity (23). Further research into postoperative neurocognitive dysfunction (PND) emphasizes that similar molecular mechanisms are involved in both PND and POCD, with aging individuals being particularly vulnerable.

Research has also explored how diabetes mellitus (DM) may contribute to POCD, as minor cognitive impairment associated with diabetes can increase the likelihood of postoperative cognitive dysfunction. Specifically, type 2 diabetes worsens early postoperative cognitive dysfunction (POCD), with diabetic patients showing more significant impairment in initial cognitive capacities compared to non-diabetic individuals after laparoscopic surgery(Seven et al., 2022b). Additionally, diabetes-induced neuropathy and vascular changes significantly impact cognitive function. These changes, which are present before and after surgery, directly impair brain structure and function. Moreover, sensorimotor neuropathy interferes with sensory information processing, which is essential for cognitive integration and processing. This increases the susceptibility of memory and executive function control centers to further damage during the perioperative period, exacerbating cognitive decline (24–26).

Vascular changes in patients with diabetes, including microvascular and macrovascular disease, significantly impact cognitive function. Hyperglycemia and insulin resistance lead to endothelial dysfunction, increased blood-brain barrier permeability, and reduced cerebral perfusion. Vascular complications such as atherosclerosis and stroke increase the risk of cognitive changes during and after surgery (27). Furthermore, chronic cerebral hypoperfusion due to vascular changes accelerates the buildup of amyloid plaques and tau hyperphosphorylation, both of which are associated with neurodegenerative diseases like Alzheimer’s and POCD study (28). A study by A. Moheet et al. clarified how diabetes affects brain structure and function, revealing that cognitive decline is linked to both type 1 and type 2 diabetes. The study emphasizes the need for comprehensive interventions to address the multifactorial nature of diabetes-related cognitive decline (29).

### 2.2 Pathophysiology of delirium in diabetic adults

Delirium, characterized by sudden disorientation, fluctuating consciousness, and impaired attention, is a common and serious postoperative complication in adult diabetic patients. Neuroinflammation, vascular restrictions, and metabolic abnormalities are complex underlying factors contributing to delirium in this population. Improper management of diabetes leads to chronic low-grade inflammation, increasing the brain’s vulnerability to perioperative stressors such as surgery and anesthesia (30).

**Figure 2:**
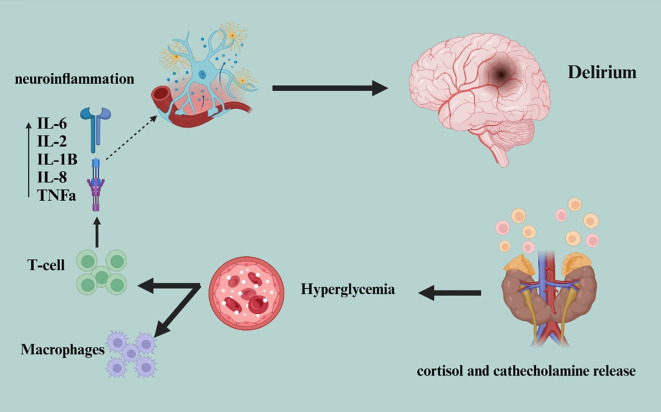
The sequence where the release of cortisol and catecholamines induces hyperglycemia, which in turn activates the immune system, triggering the release of cytokines. This cascade leads to neuroinflammation and disruptions in normal neuronal activity, ultimately resulting in the development of delirium.

Diabetes-related vascular problems, including microvascular and macrovascular disorders, limit cerebral blood flow and raise the risk of delirium and cerebral hypoperfusion. Variability in glucose levels, between hyperglycemia and hypoglycemia, aggravates cognitive problems by causing oxidative stress and disturbances in neurotransmitter production (31). Adults with diabetes are particularly vulnerable to delirium due to the combination of these factors, highlighting the need for early detection and treatment to improve postoperative outcomes (32). Kris van Keulen and colleagues’ study attempted to determine whether glucose variability is impacted during delirium versus non-delirious times in critically ill patients in the intensive care unit (ICU), both with and without diabetes. The study found that delirium and hypoglycemia are positively correlated in critically ill diabetic patients. However, delirium is not associated with greater glucose fluctuations. The findings suggest that diabetic patients with delirium should have their blood sugar levels monitored more frequently to prevent hypoglycemic episodes (33, 34). Two key features of diabetes that contribute to delirium in diabetic adults undergoing surgery are hyperglycemia and insulin resistance. Particularly during the perioperative period, hyperglycemia greatly raises the risk of delirium by encouraging the production of pro-inflammatory cytokines such as TNF-α and IL-6, which aggravate neuroinflammation (35), **Figure 2**. By crossing the blood-brain barrier and triggering microglia, these cytokines cause persistent inflammation that reduces cognitive ability.

Moreover, aggravating oxidative stress, hyperglycemia damages neurons, compromises synaptic integrity, and lowers neuroplasticity. Typical delirium symptoms, acute disorientation and concentration issues, can result from damage to hippocampus neurons necessary for memory and cognition (36).Furthermore, aggravating cerebral hypoperfusion caused by hyperglycemia is endothelial dysfunction resulting from diabetes, which increases delirium susceptibility. A feature of type 2 diabetes, insulin resistance, is clearly important in the onset of delirium. Reduced insulin sensitivity throws off the brain’s control of glucose and preservation of energy balance (37–39). This metabolic mismatch reduces glucose metabolism in the brain, therefore raising surgical sensitivity to ischemia.

Particularly, lower acetylcholine synthesis, which is essential for cognitive functions like attention, helps to explain cognitive deficits and raises the delirium risk. Rising levels of inflammatory cytokines including TNF-α, IL-6, and, which aggravate neuroinflammation and delirium, have also been associated with insulin resistance (40), **Figure 2**. Furthermore, increased free fatty acids linked with insulin resistance could cause neurodegenerative alterations, thereby increasing the risk of delirium and postoperative cognitive dysfunction (POCD) in patients with diabetes. Surgery activates the sympathetic nervous system (SNS) and the hypothalamic-pituitary-adrenal (HPA) axis, triggering the release of stress hormones like cortisol and catecholamines (41) **Figure 2**. By means of dietary changes, physical exercise, and pharmaceutical therapies (e.g., metformin and insulin sensitizers), one can improve glucose management and insulin sensitivity, therefore breaking out this vicious cycle and lowering the chance of delirium.

Improving cognitive outcomes following surgery depends on early identification of diabetes patients at risk for delirium and the application of appropriate treatments (42). The interaction of hyperglycemia, insulin resistance, and neuroinflammation greatly increases the delirium risk in diabetic patients. Reducing the risk of delirium in this vulnerable population mostly depends on efficient blood glucose control and raising insulin sensitivity. Furthermore, delirium has been associated with stress hyperglycemia. Interestingly, patients with HbA1c levels below 6.5% indicating no chronic hyperglycemia, are more likely to experience delirium, independent of their stress hyperglycemia ratio (SHR) (43). One proposed mechanism is that individuals with lower baseline glucose tolerance may experience a more pronounced physiological stress response, resulting in acute hyperglycemia that disrupts cerebral metabolism and exacerbates neuroinflammation, both of which are implicated in delirium pathogenesis.

This study suggests that the initial SHR upon admission could serve as a valuable predictor of delirium, and incorporating this parameter into clinical prediction algorithms may enhance risk stratification (44). Although associations between acute hyperglycemia, hypoglycemia, and postoperative delirium are corroborated by numerous clinical and ICU studies, the predictive significance of glucose variability indices, such as the stress hyperglycemia ratio, remains investigational and necessitates validation in extensive, prospective perioperative cohorts (45). The risk of postoperative delirium (POD) is much raised by disturbance of the neurotransmitter balance required for appropriate cognitive capacity. Particularly in those with poor blood glucose control, metabolic dysregulation, neuroinflammation, and visual alterations affect neurotransmitter systems in diabetic patients, therefore increasing their risk of delirium (46). Especially during the perioperative phase, hyperglycemia alters the production, release, and receptor-mediated actions of neurotransmitters. Particularly affecting the dopaminergic, serotonergic, and cholinergic systems, too high glucose levels produce excessive reactive oxygen species (ROS) that damage neuronal membranes and neurotransmitter pathways. Hyperglycemia reduces acetylcholine receptor sensitivity, causing cognitive problems, particularly in short-term memory and attention. Diabetes increases sensitivity to delirium resulting from cholinergic deficits and heightened glutamate excitotoxicity, which impairs neuroplasticity and neurotransmission (47).Insulin resistance significantly affects neurochemical abnormalities related to delirium. It reduces the brain’s ability to use glucose effectively, disrupting synaptic activity and neurotransmitter balance. Reduced glucose uptake by neurons leads to decreased neurotransmitter synthesis and cognitive decline. One of the most disrupted systems is the glutamatergic system, which is crucial for synaptic plasticity and learning (48). Elevated glutamate levels can lead to excitotoxicity, damaging neurons and impairing cognitive ability. Neuroinflammation, which exacerbates neurotransmitter imbalance and delirium, is often connected to the body’s response to surgical stress and diabetes. Inflammatory cytokines like TNF-α and IL-1β reduce neurotransmitter synthesis by activating microglia, impairing synaptic function and cognition (49).

Diabetes exacerbates inflammatory responses by inducing chronic low-grade inflammation, increasing the brain’s susceptibility to postoperative delirium.

Hyperglycemia, insulin resistance, and neuroinflammation all disrupt key neurotransmitter systems necessary for cognitive regulation, leading to typical delirium symptoms that impair executive function, memory, and attention (35).

Neuroinflammatory, neuronal aging, oxidative stress, neurotransmitter deficiency, neuroendocrine, diurnal dysregulation, and network disconnectivity theories have been proposed to explain delirium. These mechanisms, including neuroinflammation and neurotransmitter imbalances, are linked to delirium’s development in ICU patients (50). Additionally, recent studies suggest that delirium may be caused by disturbances in neural pathways and neurotransmitter systems. In longstanding diabetes, autonomic neuropathy, a common complication, compromises the autonomic nerves controlling physiological processes like blood pressure, heart rate variability, and vascular tone. This dysfunction can impair blood flow control, particularly in the brain, worsening cerebral hypoperfusion during surgery (51). Autonomic dysfunction exacerbates the brain’s vulnerability to delirium by impeding its ability to respond appropriately to circulatory demands during surgery. Autonomic dysfunction and neuroinflammation are closely linked in diabetic delirium. Dysfunction of the autonomic nervous system can lead to an inappropriate immune response. People with diabetes already have chronic low-grade inflammation due to insulin resistance and hyperglycemia, but autonomic dysfunction exacerbates this inflammatory state. This imbalance activates the sympathetic nervous system, which releases more pro-inflammatory cytokines, promoting neuroinflammation and increasing the risk of delirium (52) **Figure 2**.

Additionally, autonomic dysfunction impairs the brain’s ability to control its inflammatory response to perioperative stress. The parasympathetic nervous system typically helps reduce excessive inflammation, but diabetes-related autonomic imbalance impairs this process, leading to uncontrolled neuroinflammation and neuronal damage (53). A study by Jannik Stokholm et al. investigated the relationship between autonomic function and delirium, using measures such as palmar skin conductance level (SCL) and pupillometry to assess changes in autonomic nervous system activity during delirium. The study found that autonomic modulation is altered during delirium episodes in acute stroke patients and noted a higher prevalence of diabetes among those experiencing mental disturbances (54). Despite growing evidence linking diabetes to postoperative neurocognitive disorders, several limitations of the existing literature should be acknowledged. Most available studies are observational in design, limiting causal inference, and many rely on heterogeneous definitions of POCD and delirium, variable cognitive assessment instruments, and relatively short follow-up durations. Moreover, diabetic populations are frequently inadequately categorized by disease duration, glycemic control, or the existence of microvascular complications, thereby obscuring reported relationships. These constraints underscore the necessity for consistent diagnostic criteria and well-constructed prospective trials.

## 3 Factors for POCD and delirium in diabetic adults

### 3.1 Diabetes-related risk factors

Persistently high blood glucose levels, or chronic hyperglycemia, are a hallmark of poorly managed diabetes and have been identified as a significant independent risk factor for both postoperative delirium (POD) and postoperative cognitive dysfunction (POCD) (55). Prolonged hyperglycemia promotes oxidative stress, vascular dysfunction, and neuroinflammation, all of which increase the brain’s vulnerability to cognitive deterioration following surgery. In diabetic individuals, microvascular damage and reduced cerebral blood flow, both signs of chronic hyperglycemia, inhibit normal brain function and significantly worsen postoperative outcomes. Glycemic variability (GV), including episodes of both hyperglycemia and hypoglycemia, leads to the production of reactive oxygen species (ROS), which trigger inflammation and neuronal damage (56). Fluctuating glucose levels also affect the autonomic nervous system (ANS), disrupting cerebral blood flow regulation and contributing to cognitive impairment. Recent findings indicate GV may be a more predictive marker for long-term diabetic complications than traditional measures like HbA1c. GV has been linked with vascular damage and the progression of atherosclerosis, suggesting it should be a key target in diabetes management. Diabetic neuropathy, especially autonomic neuropathy, is another common complication that significantly impacts cognitive outcomes (57). Chronic hyperglycemia damages peripheral and autonomic nerves, impairing the brain’s control of blood flow and its integration of sensory information. This leads to deficits in memory, attention, and executive functioning, making diabetic patients more susceptible to POD and POCD (58). Diabetic retinopathy, though primarily affecting the eyes, reflects widespread microvascular dysfunction throughout the body, including the brain. This can compromise blood-brain barrier (BBB) integrity and reduce cerebral circulation, allowing inflammatory cytokines and neurotoxins to enter the brain more easily. This promotes neurodegeneration, especially in the hippocampus and prefrontal cortex, regions vital to memory and cognitive control (59). Emerging studies, such as those by Yiwen Li et al., have found consistent patterns among diabetic peripheral diseases (DPDs), showing shared molecular pathways and risk factors. This reinforces the need for integrated care strategies that address neuropathy, nephropathy, and retinopathy collectively, rather than in isolation (60). Early vascular interventions and stringent glucose control are essential to reduce cognitive complications. Additionally, the association between diabetic retinopathy (DR) and nephropathy (DN) suggests that DR may serve as a non-invasive predictor of broader neurological and vascular complications in diabetic individuals. Inadequate diabetes control is a key factor in the development of both POD and POCD.

**Figure 3:**
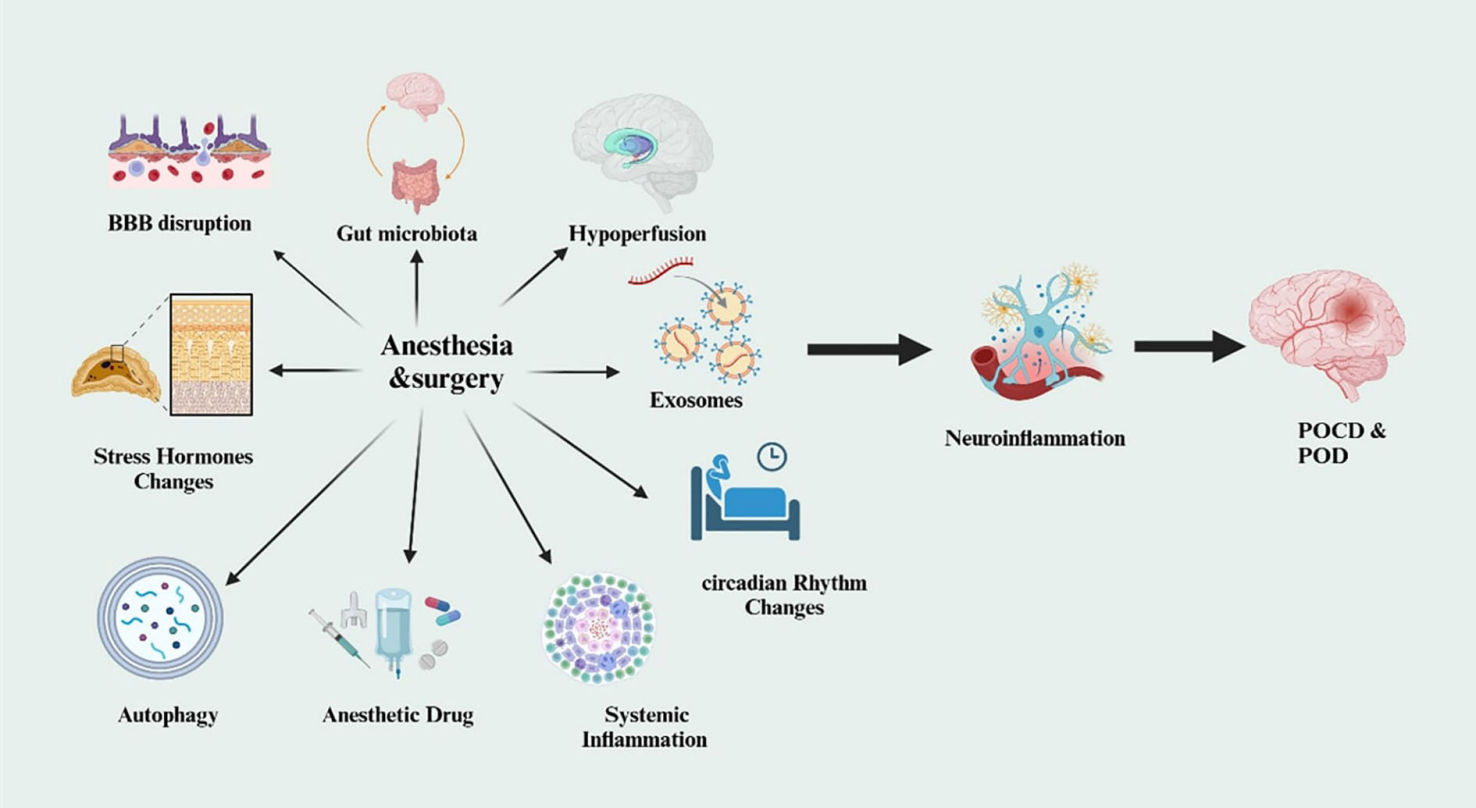
Surgery and anesthesia contribute to Postoperative Cognitive Dysfunction (POCD) and Postoperative Delirium (POD) by inducing neuroinflammation through mechanisms such as systemic inflammation, anesthetic agents, gut microbiota alterations, circadian rhythm disruption, and exosomal signaling.

Uncontrolled blood glucose whether persistently high or fluctuating induces neuroinflammation, damages cerebral vasculature, and increases BBB permeability (61) (**Figure 3**). These mechanisms impair cerebral perfusion and allow pro-inflammatory agents and toxins into the brain, thereby worsening cognitive function. During surgical stress, these effects are amplified (62). High blood sugar levels contribute to further vascular damage, impair synaptic plasticity, and elevate the risk of neurodegenerative processes. Hypoglycemia, on the other hand, can cause acute neuronal injury, especially in memory-critical areas like the hippocampus. The brain’s extreme sensitivity to glucose fluctuations underscores the importance of consistent glycemic management, particularly in the perioperative period (63).

Non-compliance with diabetes medications further exacerbates cognitive decline. Irregular glycemic control, combined with untreated vascular conditions like hypertension and dyslipidemia, compromises cerebral blood flow and increases the risk of both acute and chronic cognitive impairments. Patients undergoing surgery must maintain strict adherence to prescribed regimens to preserve cognitive function and avoid the compounding effects of neuroinflammation and vascular stress (64, 65).

### 3.2 Age and comorbidity

Particularly in patients with diabetes, aging significantly reduces cognitive resilience and increases susceptibility to postoperative delirium (POD) and postoperative cognitive dysfunction (POCD) (66). Age-related changes in brain structure and function, including decreased neuroplasticity, impaired synaptic function, and reduced neuronal regeneration, diminish the brain’s ability to recover from the stress of surgical procedures (67). As people age, the likelihood of experiencing cognitive decline, characterized by memory loss, reduced attention span, and slower processing speed, increases. These challenges are compounded by diabetes-related issues such as neuroinflammation and vascular damage (68). The ageing brain loses capacity to control the physiological demands of anesthesia and surgery and is less able to withstand these stresses. Furthermore, age-related alterations in neurovascular function reduce cerebrovascular reserve, rendering the aging brain more vulnerable to hypoperfusion, particularly during surgical procedures in which anesthesia and surgical stress can induce fluctuations in cerebral blood flow (69, 70) (**Figure 3**). In elderly individuals with diabetes, vulnerability to postoperative cognitive disorders is amplified by diabetes-related vascular and metabolic dysfunction. The interplay between age-associated neurodegeneration and persistent hyperglycemia generates a compounded risk for postoperative delirium (POD) and postoperative cognitive dysfunction (POCD). Hyperglycemia and glycemic variability induce oxidative stress, endothelial dysfunction, and neuroinflammation, leading to microvascular injury and blood–brain barrier disruption. These processes are associated with elevated inflammatory biomarkers including, IL-6, TNF-α, CRP, S100B, NSE, and impaired neuronal insulin signaling, which compromise synaptic plasticity and cognitive reserve. Neuroimaging studies further demonstrate diabetes-related structural and functional brain alterations, including increased white matter lesion burden and hippocampal atrophy, that predispose patients to perioperative cognitive impairment. (71–73). By raising neuroinflammatory responses, reducing cerebral perfusion, and increasing blood-brain barrier (BBB) permeability, glycemic swings and persistent hyperglycemia aggravate the consequences of ageing on neurovascular function. These elements render the brain less suited to the physiological demands of surgery, which causes long-term cognitive loss connected to POCD (74). Older patients with diabetes are more susceptible to unexpected cognitive problems like delirium. Furthermore, the incidence of comorbid diseases like cardiovascular disease, hypertension, and chronic kidney illness rises with ageing. These disorders further affect neurocognitive capacities and vascular integrity, therefore increasing the likelihood of cognitive problems after surgery. Older diabetic patients’ risk of delirium and POCD is much raised by age-related changes in neuroplasticity, neurovascular function, and cognitive processing (75). This is especially relevant when comorbidities connected to diabetes aggravate the condition: hyperglycemia, vascular dysfunction, and neuroinflammation. During surgery, fluctuations in blood pressure and cardiac output further exacerbate pre-existing vascular conditions, complicating the brain’s ability to maintain an adequate supply of oxygen and nutrients.

This hinders cognitive performance, particularly in regions of the brain that govern executive function, memory, and attention (76). Additionally, cardiovascular medications commonly prescribed to diabetic patients, such as beta-blockers and ACE inhibitors, may impair cognitive performance by causing electrolyte imbalances or hypotension during the postoperative phase. Renal impairment, often seen in patients with diabetes due to diabetic nephropathy, is another significant comorbidity that impacts cognitive function during the perioperative phase (77). Renal dysfunction can lead to the accumulation of uremic toxins in the bloodstream, which can cross the BBB and have neurotoxic effects on the central nervous system. Dialysis patients and those with chronic kidney disease (CKD) often experience cognitive impairment and delirium due to the elevated amounts of uremic toxins in their brains. Dialysis, while essential for renal failure treatment, can also contribute to cognitive deficits due to fluid balance changes, electrolyte imbalances, and hypotension, all of which affect brain oxygenation and perfusion (78). Hypertension and electrolyte imbalances, common in renal disease, exacerbate vascular dysfunction and cerebral ischemia, further increasing the risk of delirium and POCD. Patients with renal dysfunction also tend to have longer recovery times as their impaired ability to eliminate waste results in slower clearance of anesthetics and medications, leading to prolonged cognitive impairment after surgery.

Chronic depression, frequently associated with reduced hippocampal volume, further complicates recovery (79). Depression impairs memory and learning, both of which are critical for cognitive recovery post-surgery. Depressed patients often have poor glycemic control, contributing to elevated inflammation and an increased risk of cognitive decline (68). Additionally, depression affects the autonomic nervous system, increasing the risk of delirium and weakening cerebral blood flow regulation. Sleep disturbances and fatigue, frequently noted in individuals with depression, might intensify postoperative cognitive impairment by hindering synaptic plasticity, obstructing memory consolidation, and enhancing neuroinflammatory signaling, as indicated by increased IL-6 and TNF-α levels. Disruption of circadian rhythms and stimulation of the hypothalamic–pituitary–adrenal (HPA) axis exacerbate vulnerability to postoperative cognitive dysfunction (POCD) and postoperative delirium (POD) during the recovery phase (80–82). Consequently, the regulation of diabetes and the management of depression are crucial for enhancing cognitive results post-surgery in diabetic individuals (83).

### 3.3 Surgical and anesthetic factors

Particular surgical categories, such as cardiac and non-cardiac surgery, and the classification of surgical procedures, including major and minor surgeries, have a substantial impact on cognitive outcomes in individuals with diabetes. The development of postoperative cognitive dysfunction (POCD) and postoperative delirium (POD) is significantly influenced by these factors (84). Moreover, the complexity and severity of surgical procedures are closely linked to neurological stress responses, neuroinflammatory processes, and vascular function, all of which play a critical role in cognitive recovery (85). The type of surgery performed is a key contributing factor to these adverse outcomes, particularly in patients with diabetes, who are more susceptible to postoperative cognitive decline due to pre-existing vascular and metabolic impairments. The duration and level of invasiveness of surgical operations have a considerable impact on the severity of postoperative cognitive impairment (86). deep general anesthesia, substantial blood loss, and elevated systemic stress are associated with major surgical procedures, such as abdominal, cardiac, or vascular surgeries. These factors can worsen neuroinflammation and cerebral hypoperfusion in patients with diabetes. Surgical procedures that worsen cognitive impairment might result in increased oxidative stress, decreased cerebral blood flow, and more pronounced fluctuations in glucose levels (87). In order to reduce the risk of postoperative cognitive dysfunction (POCD) and delirium (POD) in individuals with diabetes, anesthetic techniques and pharmaceutical drugs are essential. Cognitive outcomes can be strongly impacted by the choice of anesthetic materials, the type of anesthesia (general or regional), and the management of anesthesia during the perioperative period, either improving or worsening them (88–90). Because they already have vascular impairments, neuropathy, and metabolic abnormalities, diabetic patients are more vulnerable to the effects of anesthesia. Therefore, to successfully reduce postoperative cognitive problems, it is essential to understand how specific anesthetic techniques affect the central nervous system (CNS) in individuals with diabetes. General anesthesia is commonly used after major surgery and can have a significant impact on brain function, particularly in diabetic people. Studies reveal that the use of volatile anesthetics, such as isoflurane and sevoflurane, can impair neuroplasticity and synaptic transmission (91, 92). Cerebral hypoperfusion could arise from these anesthetics’ disruption of neurotransmitter systems and reduction of cerebral blood flow (93) **Figure 3**. Compromised vascular function, which is typified by endothelial dysfunction and microvascular damage, exacerbates these effects in patients with diabetes and makes it more difficult for the brain to guarantee an adequate supply of oxygen and nutrients during surgical procedures. However, localized anesthetic techniques like nerve, spinal, or epidural blocks are linked to fewer cases of cognitive impairment during the recuperation phase (94). By eschewing the use of volatile anesthetics, regional anesthesia may reduce the effects on brain function. Localized (regional) anesthesia can help maintain cerebral perfusion by avoiding the hypoperfusion and blood pressure fluctuations commonly associated with general anesthesia (95–97). In diabetic patients, close monitoring of blood glucose levels and fluid balance remains essential even when regional anesthesia is used to support optimal cognitive outcomes. The choice of anesthetic agents also plays a critical role, as some volatile anesthetics have been linked to delirium and postoperative cognitive dysfunction (POCD), likely due to their effects on neurotransmission and neuroinflammatory pathways (98). Because of their rather constant effects on cerebral blood flow and their anti-inflammatory qualities, intravenous medicines including propofol, usually used in general anesthesia, may be linked with a lower risk of cognitive impairment. Particularly compared with other volatile anesthetics, experimental investigations indicate that propofol exhibits antioxidant and neuroprotective properties, which could help reduce the incidence of postoperative cognitive dysfunction (POCD) (99). Large clinical studies have not clearly shown its superiority, particularly in diabetic individuals who may be more prone to cognitive problems; the evidence still seems suggestive rather than decisive.

## 4 Detection and screening tools of POCD and delirium

### 4.1 Cognitive screening tools

Standardized cognitive tests help to evaluate postoperative cognitive dysfunction (POCD) in diabetic patients especially prone to cognitive decline resulting from metabolic irregularities, vascular damage, and neurological issues connected with diabetes. Importantly, POCD refers to a more subtle cognitive decline that usually develops days to weeks after surgery and may persist for months, which is different from postoperative delirium (POD), an acute fluctuating condition occurring within hours to days. These tests measure important cognitive skills including executive function, memory, processing speed, and attention, all of which could be affected following surgery especially in people with pre-existing diabetes-related problem (29). Both the MMSE (Mini-Mental State Examination) and MoCA (Montreal Cognitive Assessment) are brief cognitive screening tools used to assess cognitive function. However, referring to them as providing an insightful analysis overstates their purpose and capability. These tools were not originally designed specifically for diabetic patients, though they are often employed in broader clinical populations, including those with diabetes, due to the increased risk of cognitive impairment in this group (100). Evaluating general cognitive ability is mostly dependent on the MMSE. This quick, organized test assesses orientation, attention, memory, language, and computation among other cognitive areas. Although the MMSE is good in identifying early symptoms of dementia and POCD, especially in older diabetic patients, it may not be sensitive enough to find modest cognitive impairment (MCI) or mild cognitive changes following surgery, meaning that its sensitivity for subtle POCD can be limited, even if its specificity is higher for more advanced impairment (101). In contrast, the MoCA is generally considered more sensitive in detecting mild cognitive impairment and early postoperative cognitive decline, making it more useful for identifying subtle POCD changes. It is especially restricted in identifying executive function and processing speed deficiencies, which patients with diabetes commonly compromise. Notwithstanding its shortcomings, the MMSE is nevertheless a useful instrument for spotting notable cognitive decline in diabetes patients following surgery.

Conversely, the MoCA is a useful tool for evaluating POCD in diabetic patients since it is more appropriate for identifying small cognitive deficits and MCI (102).

Common in patients with diabetes, executive dysfunction and attentional problems are especially delicate to it. Although the MMSE provides a rapid assessment of more severe cognitive deficits, the MoCA is more skilled at spotting subtle cognitive changes and MCI particularly common in diabetes individuals. Combining both tests can provide a more whole evaluation of the cognitive condition of a diabetic patient following surgery (103). The MMSE and MoCA do, however, have limits in evaluating cognitive deterioration in diabetic individuals. As various research, particularly in populations with lower literacy levels, have shown, the MoCA, for example, may not be generally relevant across all demographic groups within the diabetic population due of cultural and educational biases (104). Moreover, although the MMSE is extensively investigated and applied, it might not be very sensitive in identifying mild cognitive decline in patients with diabetes, especially those with attention and executive function problems.

Identification of diabetic individuals at higher risk for postoperative delirium (POD) and POCD depends critically on preoperative cognitive examination. With its related metabolic, neurological, and vascular consequences, diabetes raises the risk of cognitive loss following surgery (105). Early identification of patients with pre-existing cognitive deficiencies or those at risk of developing cognitive disturbances enables personalized perioperative care, which may include cognitive rehabilitation, anesthetic changes, and glycemic control improvement. Although standard clinical tests may miss MCI or moderate executive dysfunction, careful preoperative screening can find these problems, therefore greatly affecting postoperative recovery. Commonly resulting from the metabolic and circulatory abnormalities linked with the disease, executive dysfunction and MCI are early signs of cognitive deterioration in patients with diabetes (106).

Although preoperative screening instruments like the MMSE and MoCA can assist identify people at higher risk for POD and POCD, regular clinical examinations generally overlook these cognitive abnormalities. Early identification of these hazards allows doctors to modify perioperative treatment regimens to lower the possibility of cognitive problems (4). Moreover, preserving better glycemic control is essential to avoid glycemic swings that can aggravate cognitive problems. Early cognitive screening in patients with diabetes may facilitate risk stratification and allow for targeted perioperative planning to reduce the likelihood or severity of postoperative cognitive complications such as delirium and POCD. While evidence supporting specific interventions remains limited (107). especially in diabetic populations, some studies suggest that preoperative cognitive training, optimization of glycemic control, and the use of neuroprotective strategies (e.g., minimizing sedative load, promoting early mobilization, and maintaining sleep-wake cycles) may contribute to improved short-term cognitive outcomes (108). Pharmacological approaches, such as cholinesterase inhibitors, have been explored in patients with existing cognitive impairment, but their routine use in the perioperative setting is not well supported by current evidence.

Importantly, while these measures may help mitigate cognitive decline, they have not been shown to fully prevent the long-term effects of delirium or POCD. More robust, diabetes-specific research is needed to identify effective interventions and determine whether early perioperative strategies can influence long-term cognitive trajectories or reduce the risk of future dementia (109).

### 4.2 Delirium detection tools

Especially in clinical environments, the Confusion Assessment Method (CAM) is a well-known and efficient tool for identifying delirium. Postoperative delirium (POD) is an acute neuropsychiatric syndrome that typically occurs within hours to days after surgery, characterized by fluctuating disturbances in attention and awareness. Examining key traits including acute onset disorientation, fluctuating consciousness, and inattention which are considered basic hallmarks of the condition this accelerated and comprehensive analysis seeks to identify delirium (110, 111). Renowned for its exceptional sensitivity and specificity in spotting delirium across many patient populations, including the elderly and those with chronic conditions like diabetes, the Confusion Assessment Method (CAM) Early delirium detection is made easier by the CAM, which also helps to execute suitable therapy approaches and interventions hence improving patient outcomes (112, 113). Especially in high-risk groups like those with diabetes, delirium is commonly misdiagnosed. The Delirium Rating Scale (DRS), which evaluates the degree of delirium by means of a series of questions covering cognitive and behavioral symptoms, is another instrument routinely employed. Although the DRS is less often used because of its intricacy and the evaluation time, it provides more in-depth analysis. Compared with CAM, which is widely used for rapid screening with high sensitivity and specificity, the DRS offers a more detailed severity assessment but is less practical for routine bedside application. It is, nevertheless, quite helpful in research environments when thorough observation of delirium symptoms is required. (114, 115). Tools like the 4AT (four A’s Test) have become somewhat well-known for bedside evaluation because of their simplicity and short length. Especially helpful for rapid screening in hectic hospital settings, the 4AT evaluates alertness, cognition, and attention. Although it requires more study on its application across various healthcare environments, the instrument has demonstrated great validity in spotting delirium and is straightforward for non-specialized healthcare professionals to administer (116). In patients with diabetes, variations in blood glucose levels, neuropathy, and vascular issues can lead to delirium. Confusion Assessment Method (CAM) is a rigorous approach for spotting delirium that should be included into the perioperative evaluation process. The four fundamental components of the CAM structure are changes in awareness, inattention, abrupt commencement of symptoms, and disorganized cognition (117). After surgery, diabetic individuals sometimes develop severe delirium especially if they have pre-existing cardiovascular disease or poor glucose management. Early symptom identification using the CAM and appropriate delirium treatment approaches would help to significantly increase diagnosis accuracy and reduce consequences. Because of pre-existing cognitive difficulties and postoperative stressors, patients with diabetes show increased sensitivity; consequently, the constant use of the CAM tool can help to monitor delirium beginning and advise the dosage of required drugs (118). The use of CAM in preoperative and postoperative evaluations helps early identification of delirium episodes in patients with diabetes, who can be prone to cognitive abnormalities related with glucose swings or vascular problems. Including biomarkers into the diagnostic process helps to improve delirium treatment for people with diabetes by raising diagnostic accuracy and enabling the focused drug administration (117). Important criteria for delirium in this group are inflammatory markers since neuroinflammation greatly affects cognitive impairments following surgery. Recognized in both systemic and neuroinflammatory settings, elevated concentrations of cytokines including interleukin-6 (IL-6), tumors necrosis factor-alpha (TNF-α), and C-reactive protein (CRP) have been associated to cognitive impairment. Diabetes sufferers are particularly vulnerable since hyperglycemia and insulin resistance can cause ongoing low-grade inflammation, therefore increasing the risk of inflammatory reactions following surgery (119). Studies show that first increases in inflammatory cytokines predict the development of delirium during surgical operations; increased interleukin-6 (IL-6) levels are connected to the beginning of delirium. This condition reduces the functioning ability of the brain. Characteristically of delirium, hypoglycemia may cause acute confusion, disorientation, and attentional problems.

Maintaining glucose stability is thus crucial to avoid delirium in diabetic individuals.

One good way to evaluate delirium risk is by constant glucose monitoring. This lets doctors quickly change insulin treatment and dietary control to maintain steady glucose levels and avoid cognitive problems (120). The MMSE remains a commonly used cognitive screening tool because it is quick and easy to administer. However, it lacks sensitivity in detecting mild cognitive impairment, especially among individuals with higher levels of education. In comparison, the MoCA is better equipped to identify early cognitive changes, including deficits in executive functioning, making it particularly useful in perioperative evaluations and in high-risk groups like patients with diabetes. Although both instruments serve as useful tools for preliminary cognitive screening, the MoCA tends to provide a broader assessment of subtle impairments that may influence surgical outcomes. Still, it is important to recognize that neither test is diagnostic, nor do they replace comprehensive neuropsychological evaluation. Both are also subject to limitations, including potential biases related to culture and education (121, 122).

### 4.3 Challenges in diagnosis

For those with diabetes, it might be difficult to separate delirium from pre-existing cognitive impairment and postoperative cognitive dysfunction (POCD). For those with diabetes, the vascular effects of the disease including oxidative stress, neuroinflammation, and microvascular damage often cause a pre-existing decline in cognitive performance.

These problems can compromise attention, memory, and executive ability (123). Pre-existing cognitive impairment can make it challenging to separate between continuous cognitive decline brought on by diabetes and postoperative cognitive problems brought on by surgical stress. This could complicate the way POCD and delirium symptoms are interpreted. The clinical symptoms of many diseases, including disorientation, memory loss, and attention problems, are identical and so seriously complicate the diagnosis process. In diabetic patients, especially, differentiating between delirium and postoperative cognitive dysfunction (POCD) is challenging since the symptoms of confusion, disorientation, and limited attention span match (55). Usually brought on by medical or surgical pressures, including infections or anesthesia, delirium usually manifests rapidly and is distinguished by fluctuations in consciousness. POCD and delirium, particularly in the elderly diabetic population, reveal overlapping symptoms that could cause misinterpretation or underestimation of severity, complicating diagnosis and treatment (124). Moreover, considering the frequency of pre-existing cognitive abnormalities, including early-stage vascular dementia in diabetic patients or minor cognitive impairment (MCI), it can be challenging to find cognitive decline after surgery. Subtle cognitive abnormalities, including executive dysfunction, memory loss, and reduced processing speed, indicate MCI, early dementia, and POCD. Diabetic patients have specific risk factors, including vascular damage, neuropathy, neuroinflammation, and glucose fluctuation, that raise their likelihood of developing cognitive problems following surgery (64, 125). Although conventional screening tests such as the Mini-Mental State Examination (MMSE) and the Montreal Cognitive Assessment (MoCA) are useful, they do not sufficiently identify the underlying reasons for cognitive loss in people with diabetes. Along with evaluations comprising biomarkers connected to inflammation, oxidative stress, and vascular health, a tailored diagnostic plan must include glycemic management throughout the perioperative period. Including glucose variability monitoring in diagnostic procedures would help clarify how changes in glucose levels affect cognitive outcomes and might help explain delirium or long-term cognitive damage (126). For diabetes patients, a comprehensive diagnostic program has to include multi-faceted tests assessing cognitive ability in relation to other important health criteria, including vascular health, neurological function, and glycemic control (127). MRI or CT scans are among neuroimaging modalities that should be used in tandem with cognitive evaluation tools to examine anatomical abnormalities in the brain maybe caused by diabetic vascular damage Electrophysiological markers, such EEG or evoked potential testing, could help to identify minor cognitive deficits that traditional cognitive tests would overlook and enhance the investigation of brain wave patterns (128). Before surgery, specialized protocols could help doctors identify patients at risk, facilitating comprehensive preoperative cognitive assessments and the implementation of preventive strategies, including strict glycemic control, tailored anesthesia management, and cognitive training interventions. Early diagnosis helps healthcare professionals closely monitor surgical recovery and adjust treatment plans, thereby improving cognitive function outcomes. To reduce cognitive problems, personalized therapy could call for changing anesthesia methods, applying localized anesthesia as needed, and lowering systemic inflammation (129).

## 5 Management strategies for POCD and delirium in diabetic adults

### 5.1 Preoperative interventions

Optimizing preoperative glycemic control in patients with diabetes is associated with a lower risk of postoperative cognitive dysfunction (POCD) and delirium (POD). POCD refers to subtle, longer-term cognitive decline often detected by neurocognitive testing, whereas POD presents acutely with fluctuating attention and consciousness changes, usually diagnosed using the Confusion Assessment Method (CAM). Hyperglycemia and glucose variability have been linked to oxidative stress, neuroinflammation, and vascular dysfunction, which are associated with cognitive decline after surgery. While poor glycemic control is associated with a higher risk of POD and POCD, direct causal evidence remains limited, and current findings are largely observational (4). In diabetic patients, chronic hyperglycemia and glycemic variability contribute to oxidative stress, neuroinflammation, endothelial and microvascular dysfunction, impairing synaptic plasticity and cerebral perfusion. Perioperative changes in biomarkers such as IL-6, TNF-α, CRP, and BDNF may help identify individuals at increased risk for postoperative cognitive impairment. Older diabetic adults, due to pre-existing microvascular disease, neuropathy, and glycemic instability, are particularly vulnerable to these mechanisms, which collectively increase susceptibility to postoperative cognitive dysfunction (POCD) and delirium (POD) (72, 130, 131). However, the clinical utility of these biomarkers is limited by the lack of standardized thresholds and insufficient validation in larger prospective studies.

Furthermore, it is important to keep appropriate glycemic management throughout the perioperative period since studies have connected perioperative hyperglycemia to the onset of delirium and protracted cognitive impairment. For diabetic adults, who commonly experience neuropathy, vascular issues, and glucose instability, prehabilitation is very crucial. This all-encompassing strategy improves physical and mental health before surgery, therefore enhancing recovery results and lowering the chance of cognitive disturbances (10). Key cognitive skills generally most weak following surgery in this population are memory, attention, executive function, and processing speed; hence, cognitive training within prehabilitation programs targets these areas. A mainstay of prehabilitation, physical therapy is essential for increasing mobility and lowering postoperative problems. Complications from diabetes, like neuropathy, joint stiffness, and muscular weakness, can compromise mobility and functional independence, therefore extending the recovery durations and raising the risk of delirium (132).

Preoperative physical therapy seeks to increase strength, flexibility, endurance, and balance, so helping to lower hospital stays, hasten recovery, and lower the risk of postoperative problems like infections, falls, and functional decline. This all-encompassing strategy also helps restore metabolic homeostasis, therefore stabilizing blood glucose and lowering the risk of cognitive impairment (133). Older individuals with diabetes exhibit increased susceptibility to postoperative cognitive impairment due to pre-existing microvascular disease, neuropathy, and glycemic instability. These conditions amplify neuroinflammation, oxidative neuronal injury, and neurotransmitter imbalance, ultimately compromising neural network function and increasing susceptibility to postoperative delirium and POCD. (71, 72). Commonly utilized to lower surgical stress and discomfort are several pharmacological treatments, including analgesics, sedatives, and anticholinergic medications, but they can potentially aggravate cognitive problems. Often used to reduce perioperative anxiety, sedatives can greatly affect cognitive function in diabetes patients, especially in older persons with pre-existing cognitive problems (134). Medications like barbiturates and benzodiazepines can impair memory, attention, and overall cognitive function, especially by interfering with neurotransmitter systems like gamma-aminobutyric acid (GABA), which is essential for regulating behavior and thought processes. Therefore, using the lowest effective dose and avoiding prolonged-acting sedatives is essential to minimize the risk of cognitive impairment. Alternative approaches, such as non-sedative anxiolytics and localized anesthetics, should be considered to reduce the cognitive risks of sedatives (135). Anticholinergic medications, commonly prescribed for gastrointestinal motility issues, urine retention, and preoperative sleepiness, have been associated with cognitive decline, particularly in older adults or those with existing cognitive impairments. These medications, such as scopolamine, atropine, and certain antihistamines, inhibit the neurotransmitter acetylcholine, which is critical for memory and learning. Long-term use of anticholinergics can lead to delirium, memory problems, and attention deficits.

Therefore, anticholinergic use should be minimized in diabetic patients, and when necessary, lower doses and shorter treatment durations should be preferred (136). Monitoring cognitive function during the perioperative period can also help detect early signs of cognitive decline due to anticholinergic medications, enabling timely management. Analgesics, including both opioids and non-opioid painkillers, are essential for managing postoperative pain but must be carefully managed in diabetic patients to avoid exacerbating cognitive deficits (137). Opioids such as morphine and fentanyl, commonly used to treat severe postoperative pain, can cause sedation, confusion, and delirium, particularly in older patients with diabetes at higher risk for cognitive decline.

Opioid-induced cognitive impairment, which affects memory and concentration, can also worsen delirium symptoms. Healthcare professionals should utilize the lowest effective doses of opioids and take non-opioid substitutes such as paracetamol, NSAIDs, or regional anesthesia, which are less likely to interfere with cognitive ability, to reduce these risks (138). Encouragement of opioid-sparing techniques, including nerve blocks or local anesthetics, should help to reduce cognitive hazards and guarantee efficient pain control. Furthermore, essential for controlling pain and maintaining cognitive integrity are continuous pain monitoring and postoperative mental state evaluations (139–141).

### 5.2 Intraoperative considerations

Intraoperative management might substantially affect the likelihood of postoperative cognitive dysfunction (POCD) and postoperative delirium (POD) in diabetic patients (142). Diabetic individuals are especially susceptible to cognitive problems after surgery due to pre-existing vascular dysfunction, neuropathy, and glycemic instability.

Alterations in cerebral blood flow and metabolic control generated by anesthetics have been associated with cerebral hypoperfusion and neuroinflammatory reactions, which may be linked to postoperative cognitive impairment, although the causative pathways are not fully understood (143). Mechanistic pathways underlying postoperative cognitive vulnerability in diabetic patients, including oxidative stress, neuroinflammation, and vascular dysfunction, are detailed in Section 5.1. Intraoperative factors such as anesthetic type, duration, glycemic variations, and hemodynamic instability interact with these pathways, influencing cognitive outcomes. Volatile anesthetics like sevoflurane and isoflurane can modify GABA and NMDA receptor activity in experimental models, potentially affecting synaptic transmission and neuronal excitability, but the clinical significance in humans remains uncertain (144). In animal studies, these drugs have been shown to provoke mitochondrial dysfunction, elevate ROS generation, and activate microglia, potentially leading to neuroinflammation and synaptic damage. Clinical evidence in humans is less clear (145). Human studies, however more constrained, substantiate correlations between extended exposure to volatile anesthetics, perioperative hyperglycemia, and temporary disruptions in cerebrovascular autoregulation, potentially intensifying oxidative stress and facilitating POCD (144). Conversely, intravenous anesthetics, such as propofol, exhibit antioxidant and anti-inflammatory properties by maintaining mitochondrial function and inhibiting NF-κB activation, potentially reducing certain neurocognitive hazards (146).

Intraoperative variables such as anesthetic type, duration, glycemic variations, and hemodynamic instability interact with molecular and vascular pathways, connecting perioperative care to postoperative cognitive susceptibility in diabetes patients. The use of short-acting anesthetic agents and improved recovery techniques has been suggested as a way to reduce perioperative cognitive risk; nevertheless, the existing evidence is inconsistent. (147, 148). Evidence associating inhaled anesthetics with postoperative cognitive impairment is scarce and sometimes obscured by variables such as age, comorbidities, surgical stress, and perioperative care. (144, 149). Beyond anesthesia duration, the selection of anesthetic agents may also contribute to postoperative cognitive outcomes. Intravenous anesthetics such as propofol and etomidate have been studied as alternatives. Preclinical studies suggest that propofol may have anti-inflammatory and neuroprotective effects; however, clinical evidence in diabetic patients is limited and context-dependent (144, 150). Some clinical studies suggest a possible association between propofol-based anesthesia and a lower incidence of POD or POCD; however, results are mixed and not definitive, and benefits observed in preclinical models may not fully translate to humans (151). However, other investigations, particularly those evaluating longer-term cognitive trajectories, have found no significant differences compared with alternative anesthetic regimens (152). Overall, the cognitive effects of propofol appear to be context-dependent, influenced by patient vulnerability, surgical complexity, and perioperative care, underscoring the need for further high-quality randomized trials (99, 150). Regional anesthesia procedures, such as spinal and epidural anesthesia, may reduce systemic exposure to general anesthetics, but evidence regarding their impact on cognitive outcomes in diabetic patients remains inconsistent (153). Multimodal anesthesia approaches that integrate low-dose general anesthesia with regional procedures may reduce total anesthetic exposure; however, their effects on POCD and POD in diabetic patients are not yet definitively established. In addition to anesthetic method, metabolic variables constitute a significant aspect of intraoperative cognitive risk in this demographic. Ensuring stable blood glucose levels during surgery is essential for perioperative treatment in diabetic patients.

Hyperglycemia is linked to cognitive deterioration, compromised wound healing, and immune system malfunction (154). Careful intraoperative insulin and fluid management, particularly restricting dextrose-containing fluids, when possible, may mitigate the risk of hypo- or hyperglycemia and enhance cognitive outcome. Current guidelines typically advise maintaining intraoperative glucose levels within a target range of 80–180 mg/dL. Continuous glucose monitoring and personalized insulin therapy may further diminish glycemic fluctuation; nevertheless, too stringent glucose management heightens the risk of hypoglycemia, which can negatively impact cognitive performance (155, 156). Surgical stress exacerbates vascular dysfunction, impairs glucose homeostasis, and promotes neuroinflammation, all of which are implicated in the development of postoperative delirium (POD) and postoperative cognitive dysfunction (POCD) in diabetic patients. Strategies designed to modulate the perioperative stress response have been investigated as potential methods to diminish cognitive vulnerability (157). Alpha-2 agonists, including dexmedetomidine, have been linked to a decreased incidence of delirium in certain studies; however, the data are inconsistent and context-dependent (158). Beta-blockers may be utilized in certain individuals with cardiovascular comorbidities to mitigate excessive sympathetic stimulation during surgery; however, their precise impact on postoperative cognitive outcomes remains unverified (159). Neuroimaging and electrophysiological techniques, such as EEG/ERP, fMRI, and PET, have been utilized in research to examine changes in cerebral perfusion and neural network connectivity; however, their regular intraoperative use is still constrained (73, 90, 160). The interplay of these perioperative parameters is intricate and dynamic, complicating the task of isolating the independent effects of specific intraoperative variables on postoperative cognitive outcomes. Intraoperative difficulties, including significant hemorrhage or unforeseen surgical occurrences, may extend anesthetic duration and intensify cerebral stress, thereby affecting cognitive outcomes (161). While intraoperative factors influence the risk of POD and POCD in diabetic patients, most evidence is observational, limiting definitive conclusions about causality.

### 5.3 Postoperative management and rehabilitation

Rehabilitating and managing diabetic patients after surgery is crucial for reducing the incidence of POCD and POD. It is important to note that POD typically occurs acutely within days after surgery, whereas POCD develops more gradually and may persist for weeks to months. Patients with diabetes are more vulnerable to cognitive impairments due to pre-existing vascular damage, neuropathy, and glucose fluctuations. Promoting healing and minimizing long-term cognitive damage depend on quick and forceful interventions (162). Early mobilization and cognitive rehabilitation are two main techniques that have shown good success in improving recovery and lowering cognitive deterioration in diabetic patients. After surgery, early mobilization is essential since it has been demonstrated to lower delirium and POCD risk in diabetic patients. Extended bed rest following surgery can cause joint stiffness, circulatory issues, and muscular atrophy, therefore raising the likelihood of cognitive deterioration (163). Early mobilization not only helps circulation, which is crucial for avoiding cerebral hypoperfusion, a main cause of delirium and POCD, but it also helps prevent complications, including pressure ulcers, pulmonary embolism (PE), and deep vein thrombosis (DVT).

Early ambulation also helps control blood glucose levels; therefore, improving insulin sensitivity and muscle performance should help to lower glycemic swings, aggravating cognitive problems (164). Early physical activity following surgery has been linked in studies to better cognitive recovery, higher well-being, and lower risk of delirium in patients with diabetes. Common and severe in patients with diabetes, postoperative delirium calls for a multidisciplinary approach for the best results. Because of vascular problems, glycemic swings, and neuropathy, which all affect patients with diabetes, they are more likely to develop delirium. Crucially, a thorough care plan combining pharmacological and non-pharmacological treatments is When delirium symptoms are severe, pharmacological treatments including antipsychotics like quetiapine and haloperidol often are required (165). For patients with diabetes, particularly elderly people, these drugs should be used carefully, though, since they can interfere with cognitive ability and may aggravate confusion. Low doses of antipsychotics should be given for brief periods under thorough patient monitoring.

Although benzodiazepines can be used to treat acute anxiety or sedate patients in the immediate postoperative period, their usage should be limited as their major cognitive side effects include delirium, sleepiness, and memory problems (166). For diabetes patients’ postoperative treatment to prevent delirium and POCD, fluid and electrolyte management are absolutely vital. Particularly if they have chronic renal illness, vascular problems, or autonomic neuropathy, patients with diabetes are more likely to experience fluid imbalance or dehydration following surgery. These imbalances can result in cerebral hypoperfusion, neuroinflammation, and cognitive impairment, which increase the risk of delirium and POCD (167). The goal of fluid management in the postoperative phase is to prevent dehydration and overhydration while maintaining electrolyte balance. Regular monitoring of fluid intake and output, along with serum electrolyte levels (sodium, potassium, calcium), is crucial to avoid complications such as hypovolemia, hypernatremia, or hypokalemia, which can worsen cognitive deficits.

Diabetic patients, in particular, require individualized fluid therapy based on their glucose status and renal function to mitigate the effects of fluid overload or impaired renal perfusion on brain function (168). Continuous glucose monitoring (CGM) is essential for measuring real-time glucose levels, allowing appropriate insulin changes to maintain blood glucose within the target range (80–180 mg/dL). Postoperative insulin infusion regimens or sliding-scale protocols should be tailored, particularly in insulin-treated diabetic patients. Overly aggressive insulin administration must be avoided to prevent hypoglycemia, which can aggravate neuroglycopenia and raise the risk of postoperative delirium and POCD. Glycemic swings can also be reduced by carefully choosing intravenous fluids, preferring non-dextrose-containing solutions where appropriate. Maintaining steady perioperative glucose levels through these measures enhances cognitive recovery and minimizes the likelihood of long-term postoperative cognitive impairments (169).

## 6 Outcomes and prognosis

### 6.1 Long-term cognitive outcomes in diabetic adults

Elderly individuals with diabetes are especially vulnerable to enduring cognitive deterioration post-surgery due to the combined impacts of age-related neurodegeneration and diabetes-related vascular and metabolic impairments. Chronic hyperglycemia and glycemic fluctuations induce oxidative stress, endothelial damage, microvascular dysfunction, and neuroinflammation, collectively impairing neurovascular coupling and synaptic integrity (170). These mechanisms not only increase the likelihood of POCD and POD but may also expedite the advancement toward dementia.

Biomarkers, including IL-6, TNF-α, CRP, and BDNF, in conjunction with neuroimaging indicators, can yield prognostic insights into long-term cognitive outcomes (72). It should be noted that these biomarkers are not yet standardized for routine clinical use, and their predictive accuracy requires further validation. POCD and delirium after surgery are associated with long-term cognitive deficits and may contribute to a higher risk of dementia. Improving patient outcomes and stopping more cognitive decline depend on awareness of the long-term effects of these surgical problems (171). For POCD and POD, pathophysiological processes include glycemic fluctuations, neuroinflammation, and vascular damage, all of which exacerbate diabetes in individuals. Disturbances in cerebral activity connected to POCD and POD might damage the brain and accelerate cognitive decline. Many times, diabetic patients have vascular abnormalities, including microangiopathy and decreased cerebral blood flow that aggravate their delirium and POCD. These disorders disrupt neurovascular coupling, depriving the brain of oxygen and vital nutrients, thus raising the risk of cognitive decline (4). Delirium or POCD developed during surgery could aggravate pre-existing cognitive weaknesses, hence hastening cognitive deterioration. Additionally, elderly diabetic patients who experience episodes of delirium are at a higher risk of developing dementia in the years following surgery. The long-term quality of life (QoL) of individuals with diabetes is significantly impacted by postoperative cognitive deficits, particularly POCD and POD (172). Pre-existing vascular abnormalities, glycemic fluctuations, and neuropathy can exacerbate the effects of anesthesia and surgical stress, making diabetic patients more vulnerable to cognitive problems. POCD and delirium, if not properly managed, can result in long-term deficits in mental health, physical independence, and cognitive function, which can severely reduce overall quality of life. The cognitive impairments associated with POCD and delirium can hinder executive function, memory retention, attention, and problem-solving abilities. This compounded burden of illness increases the risk of mortality, disability, and functional restrictions, thereby significantly reducing overall quality of life (173).

**Figure 4:**
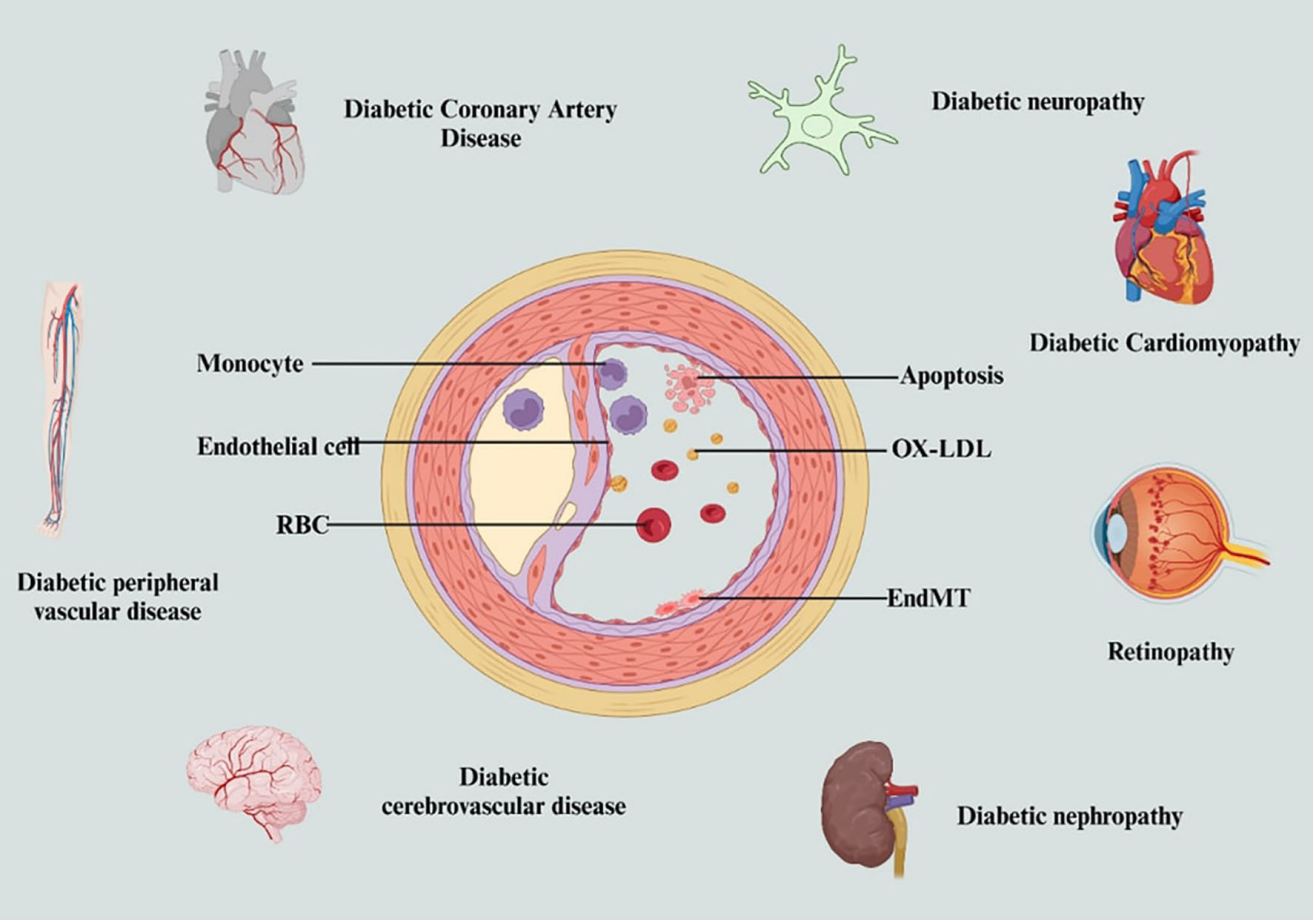
The attention to the several complicated consequences resulting from diabetes: retinopathy, cardiomyopathy, peripheral vascular disease, cerebrovascular disease, nephropathy, and so on. Important pathogenic mechanisms include the buildup of oxidised LDL (OX-LDL), death, and endothelial-to- mesenchymal transition (EndMT), which significantly affect the course of various disorders, also underlined.

Effective long-term quality of life for patients with diabetes suffering from delirium and POCD depends on early and coordinated intervention strategies. These strategies include early identification of cognitive impairments, multimodal therapies combining pharmacological and non-pharmacological treatments, and a focus on rehabilitation to support cognitive recovery (174). Early mobilization, psychological support, and cognitive rehabilitation can significantly reduce the long-term consequences of cognitive loss. Similarly, diabetic nephropathy contributes to cognitive decline by increasing the concentration of circulating toxins that can cross the BBB and interfere with brain function. Often accompanied by hypertension, nephropathy further compromises cerebral vasculature. Together with neuropathy and retinopathy, these complications collectively impair cognition, especially during the vulnerable perioperative phase (175–177). **Figure 4**. Furthermore, strict glucose control and management of vascular health are critical in improving the overall well-being of diabetic patients and slowing the progression of cognitive decline. Addressing the psychological aspects of delirium and POCD with ongoing emotional support, including therapy or mental health interventions, helps diabetic patients improve their mental health and social participation. By taking a holistic approach that accounts for both cognitive and emotional recovery, diabetes patients can significantly enhance their quality of life during the postoperative period (178).

### 6.2 Factors predicting recovery

Early identification, effective glycemic regulation, and customized rehabilitation approaches can enhance recovery from POCD and POD in diabetic individuals. While POD is characterized by sudden changes in attention and consciousness, POCD manifests as a more subtle, prolonged decline in memory, executive function, and processing speed. Cognitive recovery is affected by vascular integrity, glycemic control, and neuropathic load, with disturbances in endothelial function, chronic inflammation, and oxidative stress hindering neuroplasticity. Regular evaluation of cognitive assessments like the MMSE and MoCA, in conjunction with the monitoring of inflammatory and neurotrophic biomarkers (IL-6, TNF-α, CRP, BDNF), can inform personalized rehabilitation strategies and forecast cognitive recovery pathways (55, 105, 179).

Nonetheless, the predictive value of these biomarkers is still under investigation, and they should be interpreted cautiously in clinical decision-making. Timely intervention, optimal glycemic management, and targeted rehabilitation programs can greatly improve recovery and enhance cognitive ability. Promoting recovery in diabetes patients depends on early recognition of POCD and delirium. Usually showing up soon after surgery, delirium, which is marked by symptoms including confusion, attentional problems, and changed consciousness, typically strikes. POCD, on the other hand, could develop more quietly and show signs including delayed cognitive processing, executive dysfunction, and memory loss (30). Pre-existing cognitive problems, especially those resulting from vascular problems, may aggravate these diseases. Early identification of these problems by systematic cognitive assessments, including the Confusion Assessment Method (CAM) for delirium and instruments like the Mini-Mental State Examination (MMSE) or Montreal Cognitive Assessment (MoCA) for POCD, enables timely interventions to minimize their degree and duration (180). Early delirium and POCD diagnosis help to start customized therapy, including non-pharmacological ones such as cognitive stimulation and early mobilization, as well as pharmacological measures to control agitation. Early stages of these cognitive diseases might be subtle, particularly in diabetic patients; thus, involving a multidisciplinary team in these early phases of recovery is crucial to improve patient outcomes and increase the efficacy of treatments (181). For patients with diabetes, maintaining adequate glycemic control is critical to recovering from POCD and delirium. Blood glucose fluctuations, particularly hyperglycemia, have been associated with vascular damage, oxidative stress, and brain inflammation, which may be linked to worse cognitive outcomes after surgery (182). Poor glycemic control before and after surgery can lead to neurovascular damage, negatively affecting brain function and cerebral health. Maintaining stable blood glucose levels, ideally between 80 and 180 mg/dL, is essential for reducing glycemic variability and improving cognitive recovery. This requires a combination of oral hypoglycemic medications, postoperative insulin infusions, and continuous glucose monitoring. By avoiding periods of hyperglycemia and hypoglycemia, healthcare providers can support the healing process and enhance cognitive function (156, 183).

Rehabilitation is essential for patients recovering from POCD and delirium, particularly those with diabetes. Diabetes-related cerebral, vascular, and metabolic issues make patients with diabetes particularly susceptible to cognitive disruptions following surgery. By identifying patients predisposed to chronic cognitive impairment, healthcare providers can implement more tailored treatment strategies, improving surgical recovery outcomes. Progress in biomarker discovery and predictive modeling is enhancing the ability to detect and treat these disorders earlier (162). Predictive models, incorporating a broad range of clinical and demographic factors such as age, diabetes duration, glycemic control, vascular health, pre-existing cognitive impairments, and comorbid conditions like hypertension and renal disease, help identify patients with diabetes at higher risk for long-term cognitive impairment. Factors such as vascular diseases, advanced age, and poor glucose regulation are critical in these models. Advanced machine learning algorithms and statistical techniques are being used to develop predictive models, which analyze extensive datasets to uncover trends and risk factors that traditional clinical exams may miss (184). Models combining preoperative cognitive evaluations (like MoCA or MMSE scores), surgical complexity, and intraoperative factors (such as the type and duration of anesthesia) show great promise in predicting long-term cognitive impairment after surgery. These models provide essential information to physicians, enabling earlier interventions that could prevent or reduce the severity of cognitive impairments. The investigation of biomarkers that predict persistent cognitive impairment after surgery in patients with diabetes is a rapidly developing field (179). Biomarkers provide a more objective, sensitive, and consistent method for determining at-risk individuals. Variability in blood sugar levels and chronic hyperglycemia aggravate cognitive impairment in patients with diabetes by causing oxidative stress, visual malfunction, and neuroinflammation. Recent studies imply that biomarkers such as brain-derived neurotrophic factor (BDNF), IL-6, TNF-α, and CRP can offer insights into the fundamental processes and predict cognitive deficits post-surgery (1). Particularly important for patients with diabetes are inflammatory biomarkers since raised IL-6 and CRP levels point to increased systemic inflammation, which may increase brain vulnerability to cognitive impairment. Moreover, factors linked to a higher risk of cognitive impairment following surgery include vascular health markers, including homocysteine and fibrinogen, as well as brain damage markers, including S100B (185). Particularly in patients with pre-existing vascular problems from diabetes. These biomarkers could be early indications of POCD and delirium risk. Furthermore, offering important information for long-term cognitive prediction is research on genetic biomarkers and proteomic profiles (186). For patients with diabetes, for instance, genetic predispositions connected to neurodegenerative disorders such as Alzheimer’s and vascular dementia could raise their risk of cognitive impairment following surgery. Early warning indicators of cognitive loss could potentially be some protein markers linked to neurodegenerative illnesses, such as tau and amyloid-beta.

Though studies on these indicators are currently in progress, they have great future clinical value, especially in customized treatment for diabetic patients having surgery (187).

## 7 Future directions and research needs

It is still necessary to clarify the particular pathophysiological processes connecting diabetes with POCD and POD. Research should concentrate on strengthening diagnostic methods catered to diabetic adults and early diagnosis by means of new biomarkers.

Furthermore, randomized controlled studies evaluating the effectiveness of preoperative and postoperative therapies, including cognitive rehabilitation programs and optimal glycemic control initiatives, in lowering the prevalence of postoperative cognitive impairments in diabetic individuals.

## 8 Conclusion

Postoperative cognitive dysfunction (POCD) and delirium (POD) are serious challenges for diabetic adults undergoing surgery, often affecting recovery and quality of life. Understanding how diabetes influences these cognitive complications is essential for effective care. Early detection, personalized perioperative strategies, and targeted interventions such as careful blood sugar management, close monitoring, and cognitive rehabilitation can help reduce the risk and severity of these complications. Moving forward, research should focus on improving diagnostic methods, exploring new biomarkers, and developing innovative therapies to better protect the brains of diabetic patients during and after surgery.

## Glossary

POCD: Postoperative Cognitive Dysfunction
POD: Postoperative Delirium
CAM: Confusion Assessment Method
ERP: Event-Related Potentials
PET: Positron Emission Tomography
fMRI: Functional Magnetic Resonance Imaging
AI: Artificial Intelligence
MMSE: Mini-Mental State Examination
MoCA: Montreal Cognitive Assessment
rSO2: Regional Cerebral Oxygen Saturation
PCOS: Polycystic Ovarian Syndrome
HbA1c: Hemoglobin A1c
IL-6: Interleukin-6
TNF-α: Tumor Necrosis Factor-alpha
IL-1β: Interleukin-1 beta
BBB: Blood-Brain Barrier
CNS: Central Nervous System
ROS: Reactive Oxygen Species
DM: Diabetes Mellitus
PND: Postoperative Neurocognitive Dysfunction
T1DM: Type 1 Diabetes Mellitus
T2DM: Type 2 Diabetes Mellitus
CRP: C-reactive Protein
ANS: Autonomic Nervous System
HRV: Heart Rate Variability
SNS: Sympathetic Nervous System
PNS: Parasympathetic Nervous System
SCL: Skin Conductance Level
PLR: Pupillary Light Reflex
PCA: Posterior Cerebral Artery
ICU: Intensive Care Unit
SHR: Stress Hyperglycemia Ratio
GV: Glycemic Variability
DPN: Diabetic Peripheral Neuropathy
ESRD: End-Stage Renal Disease
CVD: Cardiovascular Disease
CKD: Chronic Kidney Disease
ACE: Angiotensin-Converting Enzyme
HRV: Heart Rate Variability
DM: Diabetes Mellitus
CABG: Coronary Artery Bypass Grafting
MCI: Mild Cognitive Impairment
MRI: Magnetic Resonance Imaging
CT: Computed Tomography
CGM: Continuous Glucose Monitoring
GABA: Gamma-Aminobutyric Acid
NSAIDs: Nonsteroidal Anti-Inflammatory Drugs
QoL: Quality of Life
BDNF: Brain-Derived Neurotrophic Factor
S100B: S100 Calcium-Binding Protein B- diabetic peripheral neuropathy
DPN: coefficient of variation of fasting blood sugar
